# THE EFFECTS OF LOSING A SECURE JOB ON HEALTH BEHAVIORS ACROSS THE LIFE COURSE

**DOI:** 10.1093/geroni/igac059.3022

**Published:** 2022-12-20

**Authors:** Qian Song, Emily Lim, Jackie Zhang

**Affiliations:** Universiy of Massachusetts Boston, Boston, Massachusetts, United States; University of Massachusetts Boston, Boston, Massachusetts, United States; Grand Valley State University, Allendale Charter Township, Michigan, United States

## Abstract

Involuntary, no-fault job loss from a relatively secure position has become more common among workers. However, it is not clear how displaced workers cope with the stress induced by the job loss and change their health behaviors due to work precarity, especially in the long-term. To address these gaps, we take advantage of the case of policy-driven layoffs from the State-Owned Enterprises in transitional China (1990s – mid-2000s), which resembles a quasi-experimental design, and examine effects of losing a secure job on dietary diversity, drinking, and smoking behaviors. We use the China Health and Nutrition Survey (1989–2015) and fixed effects models for controlling individual confounders. The results show that job loss decreases displaced workers’ dietary diversity (β=-.18, p < .05); however, the decline bounced back after three years after job loss. The career stage in which job loss occurred made a difference. The reduction of dietary diversity only applies to early-career job loss (aged 35 or younger, β=-.24, p < .1), and mid-career job loss (aged 36 – 45, β=-.37, p < .01). Job loss increases probability of drinking (OR=1.36, p < .1), but the effect is only statistically significant for late-career job loss (aged 45+, OR=1.60, p < .1). Risks of heavy drinking are only increased for early-career job losers (OR=2.09, p < .05). Risks of smoking are not impacted by job loss. The findings highlight how health behaviors serve as coping strategies for job loss, how the effects vary by the timing of job loss, and how health promotions could be helpful to address the potential risks of work displacement.

